# Vitamin D inhibits pro-inflammatory cytokines in the airways of cystic fibrosis patients infected by *Pseudomonas aeruginosa*- pilot study

**DOI:** 10.1186/s13052-019-0634-x

**Published:** 2019-03-29

**Authors:** M. Olszowiec-Chlebna, A. Koniarek-Maniecka, A. Brzozowska, A. Błauż, B. Rychlik, I. Stelmach

**Affiliations:** 1 Department of Pediatrics and Allergy, Medical University of Lodz, Copernicus Memorial Hospital, Korczak Paediatric Center, Piłsudskiego 71 Str, 90-329, Lodz, Poland; 2 Laboratory of Cytometry, Department of Molecular Biophysics, Faculty of Biology and Environmental Protection, University of Lodz, Lodz, Poland

**Keywords:** Cystic fibrosis, Calcitriol, Cholecalciferol, Interleukin 17A, Interleukin 23

## Abstract

**Background:**

Vitamin D plays an important role in inflammatory responses after antigen exposure. Interleukin-23 (Il-23) promotes Il-17-dependent inflammation during *Pseudomonas aeruginosa (P. aeruginosa)* pulmonary infection. We aimed to compare the ability of calcitriol and cholecalciferol to modulate the inflammatory response of the CF airways infected with *P. aeruginosa.*

**Methods:**

This was a randomized, placebo-controlled, double-blind, cross-over trial. Twenty-three patients with CF (aged 6–19), chronically infected by *P. aeruginosa* were randomly assigned to: **calcitriol group** receiving 1,25(OH)_2_D 0,5 mcg daily or **cholecalciferol group** receiving cholecalciferol 1000 IU daily for three months. The levels of Il-23 and Il-17A in the exhaled breath concentrate (EBC) were measured. Calcium-phosphorus balance was also evaluated (serum concentration of calcium, phosphorus, 25OHD, parathormone (PTH) and calcium/creatinine ratio in urine). Data were analyzed using means of Stata/Special Edition, release 14.2. A level of *P* < 0.05 was considered statistically significant.

**Results:**

The level of Il-17A in EBC significantly decreased in **calcitriol group** from 0,475 pg/mL (± SD 0,515 pg/mL) to 0,384 pg/mL (± SD 0,429 pg/mL) (*p = 0,008*); there was no change in **cholecalciferol group** (*p = 0,074*). The level of Il-23 in EBC did not significantly change in **calcitriol group** (*p = 0,086*); there was significant decrease in **cholecalciferol group** from 8,90 pg/mL (± SD 4,07 pg/mL) to 7,33 pg/mL (± SD 3,88 pg/mL) (*p = 0,001*). In **calcitriol group** serum phosphorus and PTH significantly decreased (*p = 0,021 and p = 0,019* respectively), the concentration of calcium significantly increased (*p = 0,001*); there were no changes in **cholecalciferol group**.

**Conclusions:**

Both analogs of vitamin D revealed their anti-inflammatory effect and reduced the level of Il-17A and Il-23 in the airway of CF patients with chronic *P. aeruginosa* infection. We observed improvement in calcium-phosphorus metabolism after supplementation with calcitriol, without adverse effects. It is recommended to use vitamin D in CF patients.

## Background

Cystic fibrosis (CF) is a chronic pulmonary disease that is associated with persistent microbial infection and chronic neutrophil infiltration. The major cause of morbidity and mortality is lung damage characterized by bronchiectasis. Chronic infection with *Pseudomonas aeruginosa* (*P. aeruginosa*) is connected with a poor clinical outcome due to persistent inflammation and production of harmful products such as proteases and oxidants secreted mainly by neutrophils and also with elevated production of pro-inflammatory cytokines such as interleukin 17A (Il-17A) and interleukin 23 (Il-23) [1, 2]. T helper 17 (Th17) cells produce Il-17A promoted by Il-23 and bind it to its receptor on the T cell membrane. They are both critical for neutrophil recruitment in a chronic *P. aeruginosa* pulmonary infection [3]. The role of the Il-17A/Il23 axis in pulmonary inflammation has been described in asthma, chronic obstructive pulmonary disease and in CF [1, 3–5]. What is more its activation correlate with inflammation status in CF individuals [1, 3, 5, 6].

1,25-dihydroxycholecalciferol (calcitriol, 1,25(OH)_2_D) is the hormonally active form of vitamin D essential for maintenance of bone and mineral homeostasis. It has been discovered to have many functions including immune system modulation [7–9]. 1,25(OH)_2_D has significant effects on function and signaling within T cell populations by downregulation monocyte toll like receptor (TLR) expression, trigger hypo-responsiveness to pathogen associated molecular patterns (PAMPs) and reduce co-stimulatory molecule and MHC II expression in monocytes and dendritic cells [9–12]. Consequently 1,25(OH)_2_D has been shown to mitigate the inflammatory effects of antigenic stimulation at epithelial surfaces [10, 11]. Patients with CF are at particular risk for vitamin D deficiency due to the high prevalence of fat soluble vitamin malabsorption, impaired hydroxylation of vitamin D and reduced sun exposure [13, 14]. This may contribute to the exaggerated inflammatory response to pulmonary infection. Most patients with CF require oral supplementation to avoid vitamin D deficiency.

We compared the ability of calcitriol - an active form of vitamin D and cholecalciferol – obtained from supplements, to modulate the inflammatory response of the CF respiratory epithelium to pathogen challenge by analysis of changes in Il-17A and Il-23 concentration in the exhaled breath concentrate (EBC).

## Material and methods

### Subjects

The total of 23 patients with CF including 11 women and 12 men between 6 and 19 years, attending the Cystic Fibrosis Outpatient Clinic in Copernicus Hospital in Lodz, Poland were included. Clinical and demographics characteristics of patients completing the study are shown in Table 1. Inclusion criteria were a diagnosis of CF, age 6 years and older, forced expiratory volume in 1 s (FEV1) above 40%, chronic *P. aeruginosa* infection (more than 50% of the monthly sputum samples positive for *P. aeruginosa* in the previous year). We excluded patients who had an exacerbation (defined by increase in symptoms, a deterioration of the FEV1 and documented radiological changes) and/or were on intravenous antibiotic therapy for at least 4 weeks before the study and/or patients with liver function abnormalities.

**Table 1:** Baseline characteristics

Variables:	Characteristic:
Age [years], mean ± SD	16,5 ± 4,38
Male gender, *n* (%)	12 (52,7)
Mutation (CFTR)	
Delta F508/other, *n* (%)	9 (39,1)
Delta F508/Delta F508 *n* (%)	10 (43,4)
Others, *n* (%)	4 (17,3)
BMI [kg/m^2^] mean ± SD	16,4 kg/m^2^ ± SD 13,43
FEV 1 [% best to predicted], mean ± SD	89,42 ± 21,02

### Study design

**Fig. 1:**
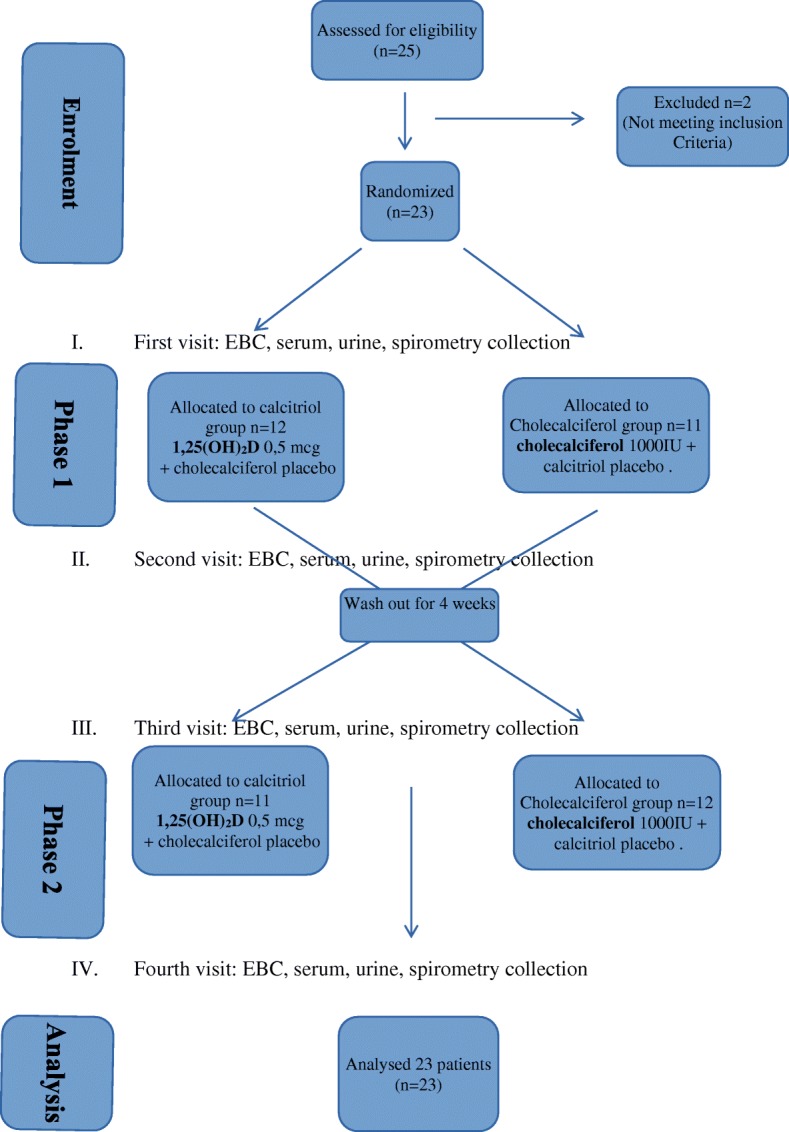
Consort diagram

This was a randomized, placebo-controlled, double-blind, cross-over trial. The study was carried out from September 2015 to April 2016. There were four study visits. At all visits, spirometry and collection of EBC took place, also serum and urine samples were collected (Fig. 1). Informed consent was obtained from all subjects and their parents and the study was approved by the local ethical committee no RNN/21/14/KE.

Eligible subjects were randomized at the first visit and divided into 2 groups of patients:

- Eligible subjects were randomized at the first visit and divided into 2 groups of patients: ***calcitriol group*** receiving: **1,25(OH)**_**2**_**D** 0,5 mcg daily in a capsule or cholecalciferol placebo in a capsule

or

- ***cholecalciferol group*** receiving: **cholecalciferol** 1000 IU daily in a capsule and calcitriol placebo in a capsule.

Randomization and treatment assignment were carried out by research pharmacy by standard allocation procedures. Only blind treatment codes were noted on randomization lists provided to study staff. All study staff and participants remained blind to treatment assignment.

The participants received calcitriol (Rocaltrol, 0,5 mcg, Roche, caps.) 0,5 mcg per day or cholecalciferol - 1000 IU per day (Bioaron D 1000, 25 mcg = 1000 IU, Phytopharm, caps.) for three months. Calcitriol, cholecalciferol and placebo were blinded by the hospital pharmacy. Patients were informed that at this time, they cannot take other vitamin D supplements. Other CF treatment according to previous recommendation was maintained. After three months (the second visit) the patients for 4 weeks returned to their standard, recommended CF therapy (in which they did not take more than 800 IU of cholecalciferol). At the third visit, according to their earlier randomization group, subjects received calcitriol 0,5 mcg per day or cholecalciferol - 1000 IU per day for the next three months. The fourth ending visit took place after 7 months from the first visit. Compliance with CF medication was checked; patients were asked to bring all used and unused medication to each follow-up visit.

### Lung function tests

Pulmonary function testing was done with a Master Screen unit (Erich Jaeger Gmbh, Hochberg, Germany). During measurements, patients were instructed to sit upright, and a nose clip and a no compressible mouth- piece were used. All pulmonary function tests were performed according to the ATS/European Respiratory Society (ERS) standards [15]. The highest of the three successful measurements was taken and analyzed. The results were expressed as the percentage of a predicted value.

### Collection and analysis of EBC

The EBC was collected by using a commercially available condenser (EcoScreen; Jaeger) according to the current ATS/ERS guidelines. Samples of the EBC were obtained from subjects during tidal breathing while wearing a nose clip. The two-way non-rebreathing valves and tubing to the condenser served as a saliva trap. After a 10-min collection, the EBC was rapidly frozen in small plastic tubes at 80 °C by using dry ice and was stored at 80 °C until analysis. All EBC samples for biochemical markers of inflammation were measured by Laboratory of Cytometry, at Cathedral of Molecular Biophysics at the University of Lodz. The level of Il-23 was conducted by using separate competitive enzyme-linked immunosorbent assay kits ELISA (ELISA Quantikine, R&D Systems) and Il-17A by using enhanced sensitivity cytometric bead array (BD™ CBAray, Biosciences). All the assays were performed according to the manufacturer’s instructions. Methods were based on literature research and the ability to quantify low concentrations of target analyzes in small volume samples.

### Laboratory tests

In serum calcium-phosphorus balance was evaluated by assessment of concentration of calcium, phosphorus, 25OHD (by a specific radioimmunoassay - 25-OH-vit.D3-RIA-CT, BioSource Europe S.A., Nivelles, Belgium), parathormone – PTH (by immunochemiluminescence assay -Intact PTH, Roche Diagnostic GmbH, Mannheim) and calcium/creatinine ratio in urine. All the above-mentioned measurements were made in the hospital laboratory using standard laboratory methods.

### Statistical analysis

The investigated traits were described by way of measures of location – mean, along with measures of dispersion – standard deviation, 95% confidence interval, and minimum-to-maximum values.

Mixed-effects regression models with robust standard errors were performed, considering both repeated measurements within the two separate studies and differences between the two studies. Intra-subject correlations also were incorporated into the mixed-effects regression models on grounds of the cross-over study design,. All the regression equations were controlled for the studied patients’ age and gender.

A level of *P* < 0.05 was considered statistically significant. All the statistical computations were carried out by means of Stata/Special Edition, release 14.2 (StataCorp LP, College Station, Texas, USA).

## Results

There were no statistically significant differences in the baseline demographics of the study groups. Both cross-over study protocols comprised the total of 23 stable CF patients – Tables 1 and 2 All 23 subjects have completed the trial.

**Table 2:** Descriptive statistics for analyzed measurable traits in the studied patients by study arm and phase of the study

Analyzed trait	Study arm	Phase of the study	Statistical parameter				Level of statistical significance (*p-value*)	
			M^a^	SD^†^	95% CI^b^	Min. – max.		
Age (years)			16,50	4,38	14,32-18,68	10–24	*a*	*b*
Phosphor (mmol/L)	Calcitriol 0,5 mcg	Before treatment	1,48	0,27	1,35-1,61	1,03-1,93	= 0,021	= 0,359
		After treatment	1,35	0,20	1,26-1,45	1,04-1,70		
	Cholecalciferol 1000 IU	Before treatment	1,53	0,23	1,42-1,64	1,05-1,87	= 0,080	
		After treatment	1,44	0,18	1,35-1,54	1,21-1,82		
Calcium (mg/dL)	Calcitriol 0,5 mcg	Before treatment	3,50	2,60	2,28-4,72	2,22-9,80	= 0,001	= 0,041
		After treatment	4,35	3,35	2,78-5,92	2,22-10,52		
	Cholecalciferol 1000 IU	Before treatment	2,81	1,62	2,03-3,59	2,22-9,48	= 0,185	
		After treatment	3,62	2,58	2,41-4,83	2,15-9,80		
Parathormone (pg/ml)	Calcitriol 0,5 mcg	Before treatment	35,64	17,61	27,40-43,88	16,99-89,12	= 0,019	= 0,208
		After treatment	29,36	13,08	23,23-35,48	9,76-72,73		
	Cholecalciferol 1000 IU	Before treatment	38,30	13,62	31,92-44,67	17,56-72,73	= 0,334	
		After treatment	34,76	16,07	26,77-42,75	17,07-89,12		
Urinary calcium/creatinine ratio (mg/dL: mg/dL)	Calcitriol 0,5 mcg	Before treatment	0,262	0,195	0,168-0,356	0,030-0,800	= 0,966	= 0,232
		After treatment	0,239	0,201	0,143-0,336	0,040-0,800		
	Cholecalciferol 1000 IU	Before treatment	0,252	0,138	0,188-0,317	0,020-0,500	= 0,050	
		After treatment	0,195	0,138	0,124-0,266	0,020-0,500		
25-OH D (ng/mL)	Calcitriol 0,5 mcg	Before treatment	24,19	8,43	20,24-28,13	5,20-40,00	= 0,125	= 0,064
		After treatment	28,19	9,75	23,63-32,76	8,10-42,90		
	Cholecalciferol 1000 IU	Before treatment	24,13	9,86	19,52-28,74	9,40-48,30	= 0,567	
		After treatment	24,97	10,86	19,89-30,06	8,00-49,00		
IL-17A in the exhaled air (pg/mL)	Calcitriol 0,5 mcg	Before treatment	0,475	0,515	0,234-0,717	0,050-1910	= 0,008	= 0,044
		After treatment	0,384	0,429	0,184-0,585	0,070-1800		
	Cholecalciferol 1000 IU	Before treatment	0,450	0,395	0,265-0,635	0,050-1910	= 0,074	
		After treatment	0,290	0,195	0,199-0,382	0,070-0,710		
IL-23 in the exhaled air (pg/mL)	Calcitriol 0,5 mcg	Before treatment	9,39	6,40	6,39-12,38	2,12-30,72	= 0,086	= 0,399
		After treatment	7,46	5,13	5,06-9,86	1,03-20,37		
	Cholecalciferol 1000 IU	Before treatment	8,90	4,07	7,00-10,80	3,04-20,72	= 0,001	
		After treatment	7,33	3,88	5,51-9,15	1,03–18,20		

(^a^*M* – mean, ^†^*SD* – standard deviation, ^b^*CI* – confidence interval. Mixed-effects regression models with robust standard errors, due to the small sample size, were performed: a – considering repeated measurements in the two separate studies; b – considering the above-mentioned repeated measurements, along with differences between the two studies and intra-subject correlation, on grounds of the cross-over study design. All statistical calculations were controlled for the studied patients’ age and sex)

### Effect of calcitriol intake on Il-17A level in EBC

**Fig. 2:**
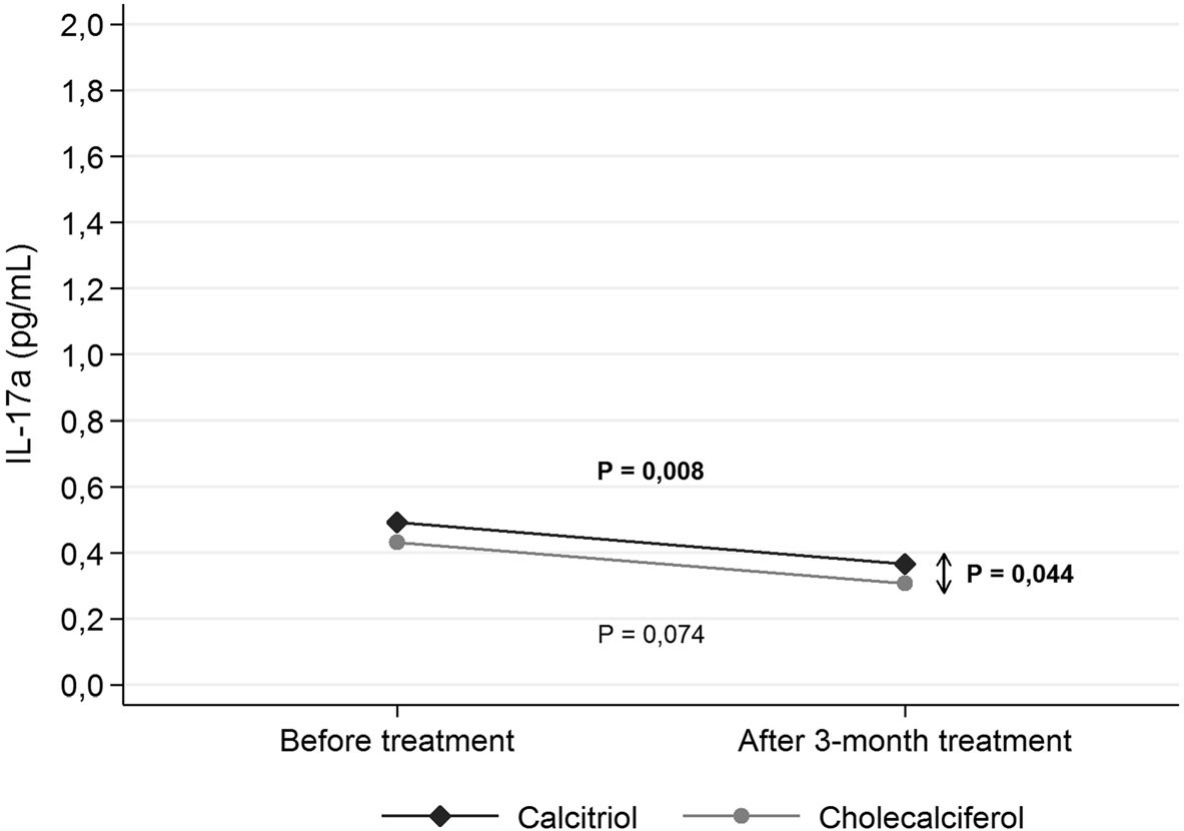
Changes in the concentration of Il-17A (pg/mL) in the exhaled breath concentrate by the studied patients before and after 3-month treatment by study arm

In the trial we study the effect of supplementation of 0,5 mcg of calcitriol on Il 17A and Il 23 level in EBC. At the end of tree months supplementation our results revealed lower level of Il-17A in EBC. It decreased from 0,475 pg/mL (± SD 0,515 pg/mL) to 0,384 pg/mL (± SD 0,429 pg/mL) (*p* = 0,008). Respectively, we did not observe change in cholecalciferol group (*p* = 0,074). The difference between the two study groups was significant (*p* = 0,044) (Fig. 2).

### Il-23 level in EBC decrease during supplementation with cholecalciferol

**Fig. 3:**
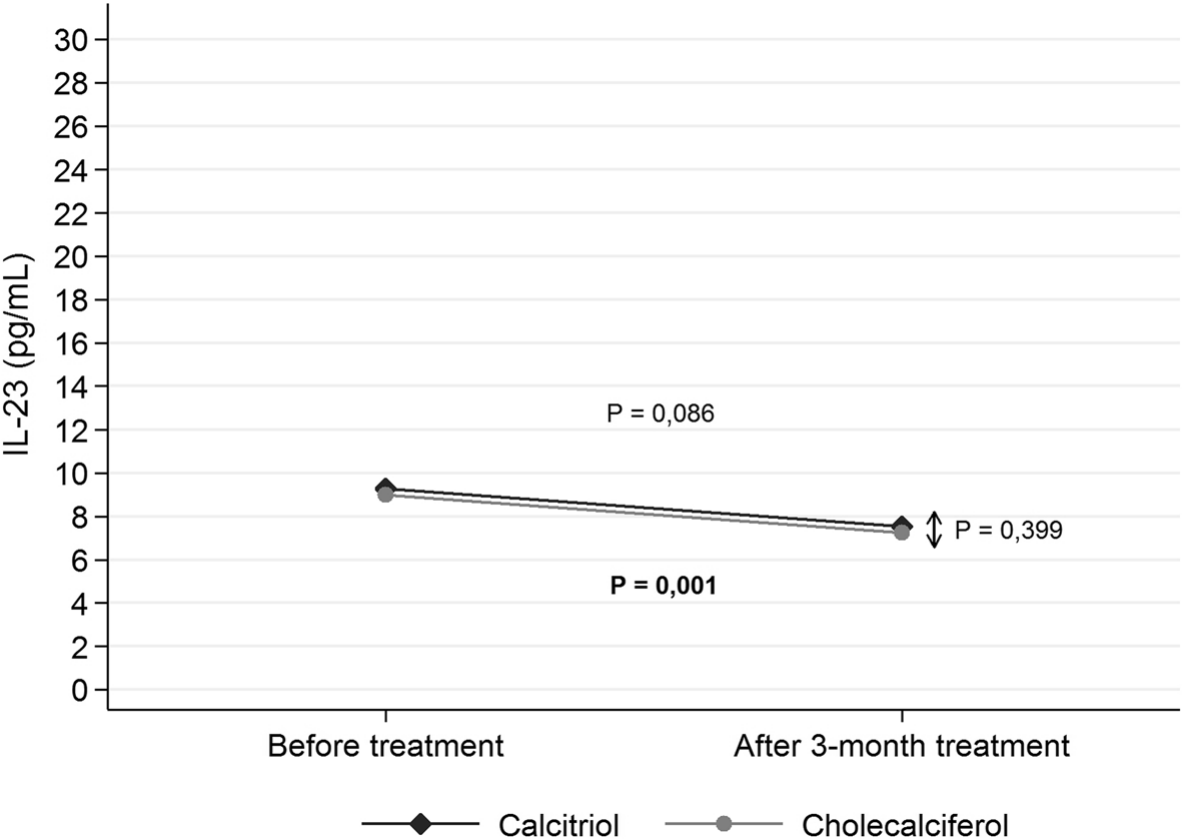
Changes in the concentration of Il-23 (pg/mL) in the exhaled breath concentrate by the studied patients before and after 3-month treatment by study arm

To evaluate the immunomodulatory effect of vitamin D in this pilot study we determined the level of pro-inflammatory cytokine: Il-23 in EBC in stabile CF patients. As expected, the Il-23 level found in EBC decreases after supplementation with 1000 IU of cholecalciferol from 8,90 pg/mL (± SD 4,07 pg/mL) to 7,33 pg/mL (± SD 3,88 pg/mL) after three months of treatment (*p* = 0,001) (Table 2, Fig. 3). Surprisingly, we did not observe changes of Il-23 level in calcitriol group.

### The effect of calcitriol and cholecalciferol supplementation on calcium-phosphorus balance

The both group of patients receiving cholecalciferol and calcitriol did not increase their 25OHD serum level. It is probably connected with low dose of cholecalciferol. Whereas the calcitriol has no direct impact on 25OHD serum concentrations. However, in calcitriol group, levels of phosphorus and PTH significantly decreased (*p* = 0,021 and *p* = 0,019 respectively) and the concentration of calcium significantly increased (*p* = 0,001) (Table 2). There was no change in the concentration of phosphorus, PTH and calcium after three months of treatment with cholecalciferol (Table 1). Urinary calcium/creatinine ratio did not change after three months of treatment in both study groups.

There were no significant changes in any of the studied clinical parameters in the both groups – BMI, FEV1.

## Discussion

The role of the immunity in the pathophysiology of CF lung inflammation with a dominant neutrophilic type of inflammation has been established [16, 17]. Several investigators have demonstrated the ability of vitamin D to modulate the inflammatory response to antigen challenge at epithelial surfaces [7, 10–12]. In this study we showed that supplementation with vitamin D might have anti-inflammatory effect. We observed that administering 0,5 mcg per day of calcitriol significantly reduced Il-17A in EBC and 1000 IU per day of cholecalciferol reduced Il-23 in EBC in CF patients with chronic *P. aeruginosa* infection. Additionally, three months treatment with calcitriol improved calcium-phosphorus metabolism and had no adverse effects, whereas cholecalciferol in administered dose did not affect calcium-phosphorus balance.

Previous studies have shown that antigen presenting cells (APC) such as dendritic cells, that are activated by bacterial antigens in the bronchial mucus layer, produce Il-23 [4, 18]. and Il-17A [19, 20]. McAllister et al [21]. found elevated Il-23 and Il-17A protein levels in bronchoalveolar lavage (BAL) and sputum of CF patients during exacerbation and decreased levels after antibiotic therapy. Other study have shown elevated levels of Il-17A and Il-23 in sputum in clinically stable but chronically infected with *P. aeruginosa* CF patients [1]. Authors suggested that in these patients Il-17A/Il-23 axis has been stimulated via activation of toll like receptor (TLR). There is evidence that vitamin D can downregulate monocyte TLR expression, triggering hypo-responsiveness to pathogen associated molecular patterns (PAMPs) [7, 10, 22]. What is more, 1,25(OH)_2_D upregulates expression and secretion of pro-inflammatory cytokines such as Il-1, Il-8 after challenge with *Pseudomonas aeruginosa* [7, 10]. Our study demonstrated that either 1,25(OH)_2_D and cholecalciferol reduced Il-17A and Il-23 in EBC in CF patients with chronical infection with *P. aeruginosa*. However surprisingly, significant Il-17A reduction in EBC was gained in calcitriol group and significant decrease in Il-23 only in cholecalciferol group*.* We speculate that cholecalciferol and calcitriol both have immunomodulatory effects but they possibly affecting different pathways with different magnitude. What is more, calcitriol is the biologically active form of vitamin D and its effectiveness is independent of the functions of the organs, while cholecalciferol supplementation could be less efficient at some CF patients especially with impaired liver or kidney function. There is known that vitamin D can inhibit Il-17A/Il-23 axis via stimulation of TLR 4 or TLR 2. It is possible that calcitriol and cholecalciferol have different effect of pro-inflammatory cytokine via different TLR pathways what may result in a interleukin expression and different activation of dendritic cells [7, 9, 12]. Additionally 1,25(OH)2D3 increase cathelicidin expression – an antimicrobial which contribute to initial defence of the airway against inhaled pathogens [10, 11]. Take both available literature and this study will not answer the question why that the statistically significant decrease of the two interleukins is found in two different treatment groups. Il-23 plays significant role in early, innate neutrophil recruitment and control Il-17 production by T cells [3]. Furthermore, Il-23 is considered as central mediator of the neutrophil inflammation seen in *P. aeruginosa* pulmonary infection. Some data have shown that neutralization of Il-23 did not result in worsening an infection [1, 3]. The results suggested that Il-23 is a promising target for the development of immunotherapeutic drugs that prevent morbidity and mortality in chronic *P. aeruginosa* pulmonary infection.

Due to calcitriol high potency, use of these vitamin D analog is associated with a higher risk of hypercalcemia and hypercalciuria compared to native vitamin D supplementation [23, 24]. In this trail we observed improvement in calcium-phosphorus metabolism in our children without adverse effects. Many studies reported that calcitriol therapy has been hampered by the common occurrence of hypercalcemia, and a great risk of renal complications in addition to their higher cost.

In our study we used calcitriol as a final product of vitamin D, which is intended for people with impaired liver and kidney function. The recommended calcitriol doses ranged from 0,25 mcg to 1,0 mcg/day as the most common dose [23, 24]. We administered the medium dose 0,5 mcg per day of 1,25(OH)_2_D in calcitriol group.

Vitamin D and its hormonally active forms are essential for maintenance of bone and mineral homeostasis. Simultaneously it has been known that vitamin D has anti-inflammatory action [7–9]. Vitamin D insufficiency occurs frequently in CF patients due to the high prevalence of fat soluble vitamin malabsorption, impaired hydroxylation of vitamin D and reduced sun exposure [13, 14]. This may contribute to the exaggerated inflammatory response to pulmonary infection. Most patients with CF require oral supplementation to avoid vitamin D deficiency but current vitamin D supplementation recommendations for CF were designed with a focus on bone health. The most trails which have shown reduction of inflammation and improved lung function in CF patient have administered at least a double dose (above 2000 IU) of cholecalciferol [25, 26]. Pincikova et al. [27]. randomized CF patients to receive 35,000–50,000 IU vitamin D per week for three months and observed that this supplementation has pleiotropic immunomodulatory effects in CF in a dose-dependent manner. Free serum 25 OH D level correlated positively with anti-inflammatory soluble immunological parameters, myeloid dendritic cells and T cell activation. We did not observe any statistically significant changes of 25OHD serum level due to the supplementation with cholecalciferol 1000 IU per day. This is probably related to low dose of administered cholecalciferol what is the limitation of our study, as we were using earlier valid guidelines [28].

## Conclusions

We demonstrated the ability of vitamin D to modulate the innate immunity and inflammatory response to antigen challenge in airway CF patients. Both analogs of vitamin D: cholecalciferol and calcitriol revealed their anti-inflammatory effect and reduced the level of Il-17A and Il-23 in EBC. Future research with vitamin D therapy is still needed and should focus on higher doses of vitamin D and using other active vitamin D analogs therapy on the inflammation status in CF patients.

## Abbreviations

1,25(OH)_2_D: 1,25-dihydroxycholecalciferol (calcitriol)
CF: Cystic fibrosis
EBC: Exhaled breath concentrate
Il-17A: Interleukin 17A
Il-23: Interleukin 23
*P. aeruginosa*: *Pseudomonas aeruginosa*
PTH: Parathormone

## References

[1] Decraene A, Willems-Widyastuti A, Kasran A, De Boeck K, Bullens DM, Dupont LJ (2010). Elevated expression of both mRNA and protein levels of IL-17A in sputum of stable cystic fibrosis patients. Respir Res.

[2] Bullens DM, Decraene A, Seys S, Dupont LJ (2013). IL-17A in human respiratory diseases: innate or adaptive immunity? Clinical implications. Clin Dev Immunol.

[3] Dubin PJ, Martz A, Eisenstatt JR, Fox MD, Logar A, Kolls JK (2012). Interleukin-23-mediated inflammation in Pseudomonas aeruginosa pulmonary infection. Infect Immun.

[4] Bullens DM, Truyen E, Coteur L, Dilissen E, Hellings PW, Dupont LJ (2006). IL-17 mRNA in sputum of asthmatic patients: linking T cell driven inflammation and granulocytic influx?. Respir Res.

[5] Dubin PJ, McAllister F, Kolls JK (2007). Is cystic fibrosis a TH17 disease?. Inflamm Res.

[6] McAllister F (2005). Role of IL-17A, IL-17F, and the IL-17 receptor in regulating growth-related oncogene-alpha and granulocyte colony- stimulating factor in bronchial epithelium: implications for airway in- flammation in cystic fibrosis. J Immunol.

[7] McNally P, Coughlan C, Bergsson G, Doyle M, Taggart C, Adorini L, Uskokovic MR (2011). Vitamin D receptor agonists inhibit pro-inflammatory cytokine production from the respiratory epithelium in cystic fibrosis. J Cyst Fibros.

[8] Grzelak T, Mikołajczyk K. Pleiotropic effect of vitamin D in cystic fibrosis. Adv Respir Med. 2018;(8):15. 10.5603/ARM.a2018.0029 30110122

[9] Wani WA, Nazir M, Bhat JI, Malik EU, Ahmad QI, Charoo BA, Ali SW. Vitamin D status correlates with the markers of cystic fibrosis-related pulmonary disease. Pediatr Neonatol. 2018;(7):19. 10.1016/j.pedneo.2018.07.001 30093293

[10] Yim S, Dhawan P, Ragunath C, Christakos S, Diamond G (2007). Induction of cathelicidin in normal and CF bronchial epithelial cells by 1,25-dihydroxyvitamin D(3). J Cyst Fibros.

[11] Sadeghi K, Wessner B, Laggner U, Ploder M, Tamandl D, Friedl J (2006). Vitamin D3 down-regulates monocyte TLR expression and triggers hyporesponsiveness to pathogen-associated molecular patterns. Eur J Immunol Feb.

[12] Penna G, Adorini L (2000). Alpha,25-dihydroxyvitamin D3 inhibits differentiation, maturation, activation, and survival of dendritic cells leading to impaired alloreactive T cell activation. J Immunol Mar.

[13] Moustaki M, Loukou I, Priftis KN, Douros K (2017). Role of vitamin D in cystic fibrosis and non-cystic fibrosis bronchiectasis. World J Clin Pediatr.

[14] Pincikova T, Paquin-Proulx D, Sandberg JK, Flodström-Tullberg M, Hjelte L (2017). Clinical impact of vitamin D treatment in cystic fibrosis: a pilot randomized, controlled trial. Eur J Clin Nutr.

[15] Miller MR, Hankinson J, Brusasco V (2005). ATS/ERS task force. Standardisation of spirometry Eur Respir J.

[16] Margaroli C, Garratt LW, Horati H, Dittrich AS, Rosenow T, Montgomery ST, Frey DL, et al. Elastase exocytosis by airway neutrophils associates with early lung damage in cystic fibrosis children. Am J Respir Crit Care Med. 2018;(10):3. 10.1164/rccm.201803-0442OC PMC6444666 30281324

[17] Roesch EA, Nichols DP, Chmiel JF (2018). Inflammation in cystic fibrosis: An update. Pediatr Pulmonol.

[18] Taylor PR, Bonfield TL, Chmiel JF, Pearlman E (2016). Neutrophils from F508del cystic fibrosis patients produce IL-17A and express IL-23 - dependent IL-17RC. Clin Immunol.

[19] Hsu D, Taylor P, Fletcher D, van Heeckeren R, Eastman J, van Heeckeren A, Davis P (2016). Interleukin-17 pathophysiology and therapeutic intervention in cystic fibrosis lung infection and inflammation. Infect Immun.

[20] Bayes HK, Ritchie ND, Evans TJ (2018). Interleukin-17 is required for control of chronic lung infection caused by Pseudomonas aeruginosa. Infect Immun.

[21] McAllister F, Henry A, Kreindler JL, Dubin PJ, Ulrich L, Steele C (2005). Role of IL-17A, IL-17F, and the IL-17 receptor in regulating growth-related oncogene-alpha and granulocyte colony-stimulating factor in bronchial epithelium: implications for airway inflammation in cystic fibrosis. J Immunol.

[22] Ashenafi S, Mazurek J, Rehn A, Lemma B, Aderaye G, Bekele A, Assefa G, et al. Vitamin D_3_ Status and the Association with Human Cathelicidin Expression in Patients with Different Clinical Forms of Active Tuberculosis. Nutrients 2018(6)4;10(6):721 10.3390/nu10060721 PMC6024873 29867045

[23] Peppone LJ, Hebl S, Purnell JQ, Reid ME, Rosier RN, Mustian KM, Palesh OG, et al. The efficacy of calcitriol therapy in the management of bone loss and fractures: a qualitative review. Osteoporos Int. 2010;(7):1133–49. 10.1007/s00198-009-1136-2 PMC3063996 19960185

[24] Tanakol R, Gül N, Üzüm AK, Aral F. Calcitriol treatment in patients with low vitamin D levels. Arch Osteoporos 2018(10)23;13(1):114. 10.1007/s11657-018-0529-2 30353299

[25] Simoneau T, Sawicki GS, Milliren CE, Feldman HA, Gordon CM (2016). A randomized controlled trial of vitamin D replacement strategies in pediatric CF patients. J Cyst Fibros.

[26] Dauletbaev N, Herscovitch K, Das M (2015). Down-regulation of IL-8 by high-dose vitamin D is specific to hyperinflammatory macrophages and involves mechanisms beyond up-regulation of DUSP1. Br J Pharmacol.

[27] Pincikova T, Paquin-Proulx D, Sandberg JK, Flodström-Tullberg M, Hjelte L (2017). Vitamin D treatment modulates immune activation in cystic fibrosis. Clin Exp Immunol.

[28] Walkowiak J, Pogorzelski A, Sands D, Skorupa W, Milanowski W, Nowakowska A, et al. Rules for the diagnosis and treatment of cystic fibrosis/Recommendations of the Polish Society of Cystic Fuibrosis 2009. Standardy Medyczne Pediatria. 2009(6):352–78.

